# Heavy metal contamination assessment and probabilistic health risks in soil and maize near coal mines

**DOI:** 10.3389/fpubh.2022.1004579

**Published:** 2022-10-13

**Authors:** Xiujuan Yang, Bijun Cheng, Yi Gao, Hongmei Zhang, Liangpo Liu

**Affiliations:** ^1^Department of Public Health Laboratory Sciences, School of Public Health, Shanxi Medical University, Taiyuan, China; ^2^Academic Affairs Office, Shanxi Medical University, Taiyuan, China; ^3^Department of Toxicology, School of Public Health, Shanxi Medical University, Taiyuan, China; ^4^Department of Environmental Health, Shanxi Medical University, Taiyuan, China

**Keywords:** probabilistic health risk, heavy metal, soil, maize, Monte Carlo simulation, coal mines

## Abstract

**Objective:**

Coal mining activities have continuously introduced heavy metals into the soil–crop system, causing increasing damage to crops. This study integrated the analysis of the heavy metal contamination status and human health risk in soil and maize near coal mines to help formulate control strategies for soil quality, maize production, and safe consumption.

**Method:**

This study was carried out on maize agricultural land near a coal mining plant. Heavy metal contamination was assessed by the geo-accumulation index (I_geo_), enrichment factor (EF), and bioaccumulation factor (BCF). The Monte Carlo simulation was used to estimate the probabilistic health risk of heavy metals exposure in soil and maize. The relationship between the concentration of heavy metal in the soil and that in maize was further visualized by correlation analysis and random forest analysis.

**Results:**

The results revealed that the mean concentrations of soil Ni, Cu, As, Cd, Sn, Zn, Pb, and Hg were all above the local background level. Ni was the most severely polluted heavy metal in maize and had a concentration higher than the risk control standard for corn in China (NY 861-2004). The I_geo_ values of all heavy metals were low, and EF values showed enrichment in V, Cr, Ti, Ni, and As. The assessment of probabilistic health risk exposed by heavy metals in soil and maize indicated that 1.16 and 1.46% of residents exceeded the carcinogenic risk level due to heavy metal exposure from soil and maize, respectively. Children were the most sensitive to maize and soil heavy metal exposure in the contaminated area. Ingestion of heavy metals was associated with the highest health risk to residents, followed by dermal contact and inhalation. As and Cr in soil and Cr and Ni in maize had the greatest impact on human health risk. Furthermore, maize heavy metals were affected the most by soil Cr, Cd, and V.

**Conclusion:**

These results may provide useful information for human carcinogenic risk associated with soil and maize heavy metal exposure due to coal mining activities.

## Introduction

Soil heavy metal pollution has become an increasing problem with the expansion of urbanization, industry, and agriculture (1). In 2020, the China Ecological Environment Status Bulletin identified heavy metals as the main pollutant of soil, stating that heavy metals, primarily Cd, adversely affect the environmental quality of agricultural land (2). Extensive human activities, such as application of pesticides, metal smelting, mining, sewage irrigation, and transportation, have released huge quantities of heavy metals into the soil ecosystem (3). Non-biodegradable heavy metals can store in the soil over time, eventually reaching concentrations that exceed safe limits and exhibiting adverse effects on plant physiological functions and human health (4, 5). Zhou (6) analyzed heavy metal pollution survey data from cultivated land in China between 2008 and 2018 and reported that most severe heavy metal pollution was caused by Cd, which had an average geo-accumulation index (I_geo_) of 0.63, followed by Hg and Ni. In Hanzhong, China, Cd and As had higher mobility than other heavy metals, and their average concentrations in rice were mildly above than the acceptable threshold (7).

Humans can be exposed to heavy metals in various ways, such as consumption of food crops, direct soil ingestion, dust inhalation, and dermal contact (8). Soil heavy metals pose severe risks to soil function and human health through the food chain (9). In addition, heavy metals in crops cause severe and destructive impacts on human health owing to long-time intake (10). Some heavy metals, such as Cd, Pb, Cu, and Cr, may affect the liver, central nervous system, kidneys, and mental health (4). In maize harvested in northern Ningxia, the content of Pb and Cr surpassed the standards (NY 861-2004) and posed non-carcinogenic and carcinogenic risks to 0.62 and 8.23% of residents, respectively (11). In Zambia, the non-carcinogenic risk values for Pb and Cd were much higher than 1, suggesting that people who consume corn grains might be at high risk of exposure to toxic levels (12). Therefore, it is necessary to assess the heavy metal pollution of soil and crops and the associated human health risks to address the concerns of public health and environmental quality.

Previous studies have estimated health risks with health risk model developed by the United States Environmental Protection Agency (US EPA), which is recognized as the most holistic approach for manifesting environmental contaminant risks (3, 13–15). However, the model's deterministic assessment focuses on the total heavy metal concentrations and exposure parameters, neglecting the significant differences among individuals and the dynamics, variability, and randomness of the heavy metal exposure process (16). The Markov Chain Monte Carlo simulation is often used to provide accurate and practical assessment of complex environmental pollution, and it has a good ability to discern the greatest influence parameter on health risks (1). Therefore, it is necessary to utilize the Monte Carlo simulation to control for the differences, lack of comparability, and inaccuracy of health risk assessment.

Heavy metals from polluted agricultural soil can be transmitted by crop root accumulation to grains, and a relationship between heavy metal concentrations of the soil and those of crops was identified (17). Research in the northern region of Malaysia demonstrated that Cu was more mobile than As, Cr, Pb, and Cd from the soil to the roots of paddy plants, and the bioavailability of Cd from the soil to the roots was poor because of poor mobility (18). The different degrees of heavy metal concentrations might be caused by the variation in crops' absorption capacity for different soil heavy metals (17). Liu et al. (11) found that in maize from northern Ningxia, China, Cd had the highest accumulation ability, and Pb had the lowest. According to Wei et al. (13), Zn, Cd, Cr, and Cu are easily enriched in maize, and Zn could be quickly translocated into the aerial part of maize and ultimately accumulated in the grains. Vegetables harvested from farms contaminated with heavy metals in western Nigeria had a greater capacity to absorb Cd and Pb than Cr, and Zn (15). Moreover, the average bioaccumulation capacity of Cd was greater than that of other heavy metals in grape pulp from a vineyard in Henan Province (3). Further research showed that Cd had a greater ability for translocation than Zn and Pb in maize cultivated in Zambia (12). The mutual influence between multiple soil heavy metals makes it necessary to explore the relationships between various heavy metals in the soil–crop system.

Shanxi Province is a representative region of prosperity and development in China, and it is an crucial industrial province because of its high coal production. However, in recent decades, rapid industrial development has led to environmental problems, causing major concern. Previous research reported serious pollution of cultivated land in Shanxi Province (6). However, rare studies have explored the heavy metal contamination of soil and plants in Shanxi Province. Therefore, it is necessary to access the ecological risks caused by heavy metals and the potential health risk to inhabitants of Shanxi Province.

This study aimed to (i) evaluate the pollution characteristics of heavy metals in agricultural soil and the harvested crops around a coal mining area; (ii) assess the probabilistic human health risk with heavy metal exposure in agricultural soil and its harvested crops; and (iii) evaluate the relationships between the concentrations of heavy metal in the soil and those in crops.

## Materials and methods

### Site description and soil sampling

We selected Jinzhong City (Shanxi Province, Northern China) as the investigation area. The study area is a warm, temperate, semiarid continental monsoon climate region, which is characterized by hot, rainy summers and generally cold, dry winters. The city of Jinzhong (36°40'−38°06' N, 111°25'−114°05' E), located in the hinterland of Shanxi Province, China, has moderate climate conditions with average temperatures between 4.2 and 14.2°C and a wide variety of soils, making it a suitable area for crop growth. Because of the unique climate and soil conditions, the local geoponics is prosperous, which has led to huge inputs of fertilizers and pesticides. Additionally, the light industry is the dominant industry in this district, and other diversified industries, including machinery, metallurgy, electrical, chemical, coal, light textile, building materials, and food, are also present. However, the expansion of coal mining and increased industrial activity pose a growing severe threat to soil quality and crop safety because of heavy metal pollution.

**Figure 1:**
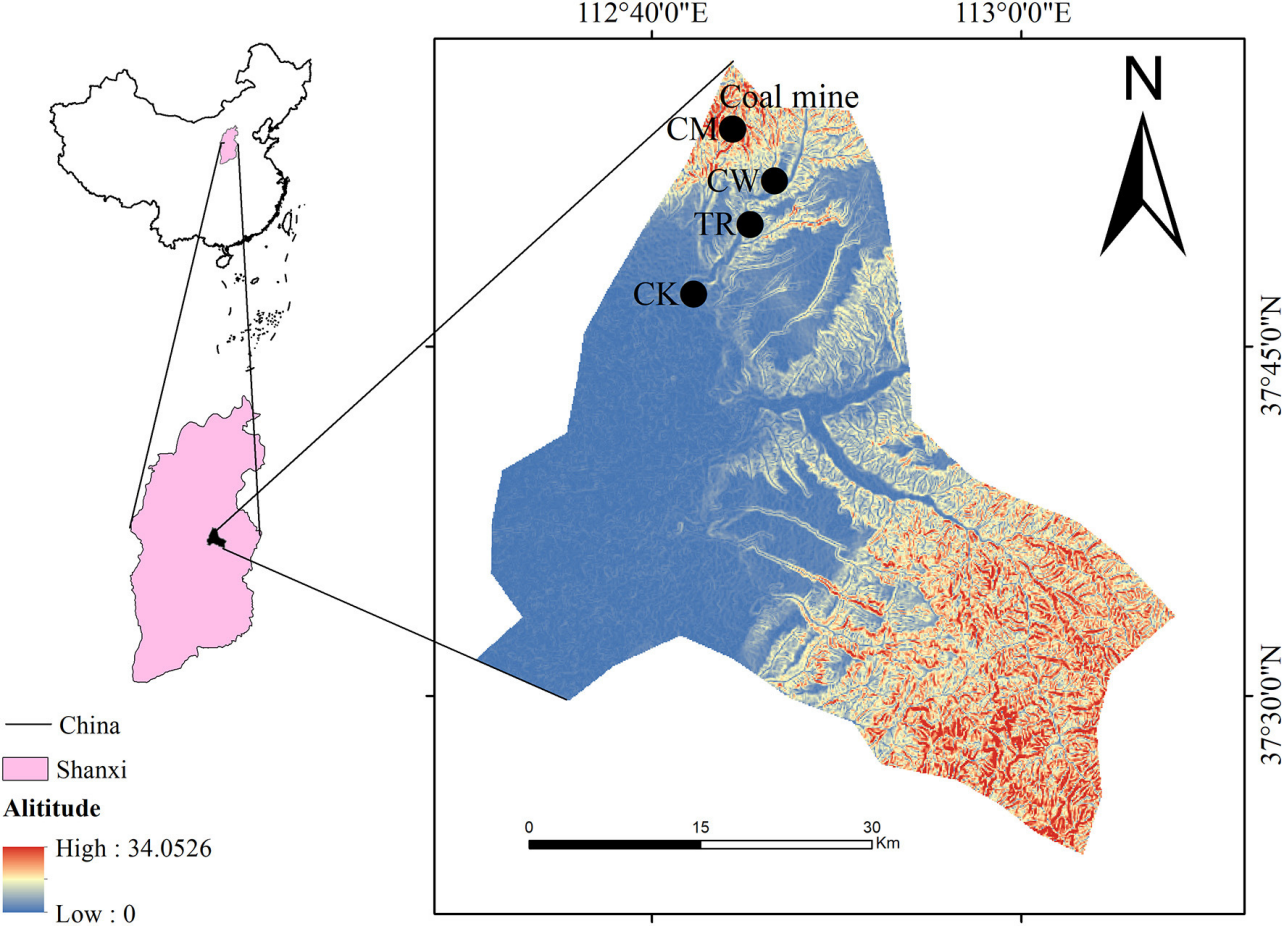
Soil and maize sampling sites in the Jinzhong City, Shanxi Province, Northern China. The Beishan coal mining plant, CM; the coal washing plant near the Hanshan coal mining area, CW; the coal transportation road, TR; the control area, CK.

The study was conducted in coal mining areas in Shanxi Province, namely the Beishan coal mining plant (CM), the coal washing plant near the Hanshan coal mining area (CW), and the coal transportation road (TR, Figure 1). The control area (CK) was located away from the coal mining areas and had no industrial influence. A random sampling method was used to select 36 paired samples of soil and maize from each study area, and each of the 36 soil and maize samples was a well-mixed composite of 9 subsamples. The nine subsamples were sampled by the systematic grid method. Approximately 3 kg of topsoil subsamples (from a 0–20 cm depth) were sampled from each site by a stainless-steel shovel. Soil samples and their corresponding crop samples were collected at the same sites during the harvest season in October, 2020. We chose maize as the representative crop because it was the main crop in the investigated areas.

### Analysis of heavy metal pollution status of soil and maize

The concentrations of nine heavy metals (Cr, V, Ni, Pb, As, Ti, Cd, Cu, and Zn) in the soil and crop samples were obtained. After sampling, the crop samples were dried at 105°C for 1 h and then dried to constant weight at 70°C. Subsequently, 300 g of crop samples were comminuted and ground, passed through a 100-mesh nylon sieve, and then packed in airtight polyethylene bags for further analysis and storage. The crop samples were microwave-digested, and the total concentrations of heavy metals were determined by inductively coupled plasma mass spectrometry (ICP-MS) using an Agilent 8800 (Agilent Technologies, Tokyo, Japan) (19). The soil samples were air-dried to a constant weight, ground, and passed through a 100-mesh nylon sieve. Subsequently, 0.2 g of dry soil samples were microwave-digested, and the heavy metal concentrations were determined by ICP-MS (19). Recovery for the analyzed heavy metals ranged from 90 to 115%, and the accuracy of the standard deviation for duplicate samples was within 10%. The entire process complied with the quality requirements of the Chinese National Standard HJ/T 166-2004, Soil Environmental Monitoring Technical Specifications.

The I_geo_ and enrichment factor (EF) were applied to evaluate the contamination status of heavy metals in the soil (1). The I_geo_ is widely used to evaluate the contamination degree of heavy metals; it compares the measured concentration and background value in the following equation:


(1) $$\begin{array}{l} I_{\text{geo}}=\log_{2}\left(\frac{C_{i}}{1.5\times B_{i}}\right) \end{array}$$

where C_i_ and B_i_ are the concentrations of each element in the sample and the background soil, respectively. The heavy metal concentrations in the CK area were regarded as background values.

The EF can be used to comprehensively compute contamination due to multiple heavy metals, and it can be evaluated by the following equation (20):


(2) $$EF=(\frac{C_{i}}{C_{ref}})/(\frac{B_{i}}{B_{ref}})\;$$

where C_ref_ and B_ref_ are the concentrations of a reference element in the sample and background soil, respectively. Mn was used as the reference element because of its stability in the soil and the lower coefficient of variation (CV) (Table 1).

**Table 1:** Statistical characteristic of the heavy metal concentrations (mg/kg) in the soil and maize of the study area.

**Heavy metal**	**Means ±SD (range)**	**CV**	**CK**	**Background**	**Screening value**
**Soil**					
V	45.13 ± 2.51 (37.14–40.79)	0.06	35.67	63.4	–
Cr	46.74 ± 6.7 (28.72–36.37)	0.18	29.94	55.3	250.00
Ti	562.5 ± 46.8 (405.9–487.99)	0.10	371.58	4000	–
Ni	30.76 ± 1.66 (25.29–27.56)	0.06	23.08	29.9	190.00
Cu	24.32 ± 1.24 (19.52–21.19)	0.06	23.16	22.9	100.00
Zn	88.7 ± 7.22 (62.78–72.17)	0.10	77.26	63.5	300.00
As	14.23 ± 0.75 (11.64–12.58)	0.06	11.45	9.1	25.00
Cd	0.39 ± 0.06 (0.2–0.26)	0.23	0.26	0.1	0.60
Pb	19.65 ± 1.34 (15.5–17.49)	0.08	21.09	14.7	170.00
Mn	564 ± 13.37 (517.2–542.21)	0.02	504.83	532	–
**Maize**					
V	0.0138 ± 0.0068 (0.004–0.022)	0.4906	0.0307	–	–
Cr	0.56 ± 0.67 (0.09–1.5)	1.19	0.28	–	1.00
Ti	6.76 ± 2.28 (3.62–9.15)	0.34	6.56	–	–
Ni	0.61 ± 0.19 (0.35–0.82)	0.31	0.31	–	0.40
Cu	2.86 ± 1.46 (1.53–5.33)	0.51	1.92	–	10.00
Zn	24.73 ± 3.58 (20.61–30.37)	0.14	20.40	–	50.00
As	0.0167 ± 0.0149 (0.002–0.039)	0.8955	0.0220	–	0.70
Cd	0.0026 ± 0.0011 (0.0005–0.0038)	0.4077	0.0021	–	0.05
Pb	0.0664 ± 0.0676 (0.018–0.193)	1.0170	0.0540	–	0.20
Mn	6.72 ± 1.79 (4.63–9.09)	0.27	5.27	–	–

The bioaccumulation factor (BCF) severed as an evaluation index to assess the extent of heavy metal contamination in maize (13). It was calculated by the following formula:


(3) $$\begin{array}{l} BCF=\frac{C_{maize}}{C_{soil}} \end{array}$$

where C_maize_ and C_soil_ are the concentrations of heavy metals in the maize and the soil, respectively.

### Probabilistic health risk assessment of soil and maize

Health risk assessment was used to evaluate and predict the probability of the occurrence of adverse effects in humans, including non-carcinogenic and carcinogenic risks (21). Heavy metals can impact the human body after dermal contact with soil (der), expiratory inhalation of soil particles (inh), and oral ingestion of soil dust (ing) (19). In this study, the human exposure risk assessment method was used to evaluate the health risk of heavy metals. The model of exposure and parameters referred to the US EPA Exposure Factors Handbook: 2011 Edition, and the exposure factors of skin and average body weight were modified according to the technical guidelines for risk assessment of contaminated sites (HJ25.3-2014), the technical guidelines for deriving soil environmental criteria for human health (draft for comment) (2018), and related studies (3, 8, 21). The probabilistic health risk was calculated by the following formulas:


(4) $$\begin{array}{l} ADD_{ing}=\frac{C_{i}\times IngR\times CF\times EF\times ED}{BW\times AT} \end{array}$$

(5) $$\begin{array}{l} ADD_{der}=\frac{C_{i}\times CF\times SA\times AF\times ABS\times EF\times ED}{BW\times AT} \end{array}$$

(6) $$\begin{array}{l} ADD_{inh}=\frac{C_{i}\times IngR\times EF\times ED}{PEF\times BW\times AT} \end{array}$$

(7) $$\begin{array}{l} HQ_{i}=\frac{ADD_{i}}{RfD_{i}} \end{array}$$

(8) $$\begin{array}{l} HI=\overset{\text{n}}{\underset{i=1}{\sum }}HQ_{i} \end{array}$$

(9) $$\begin{array}{l} CR_{i}=ADD_{i}\times SF_{i} \end{array}$$

where ADD_ing_, ADD_der_, and ADD_inh_ represent the average daily doses of heavy metals in the soil and maize in mg/(kg·d); HQ_i_ is the hazard quotient used for estimating the non-carcinogenic effects of the ith heavy metal on a specific exposure pathway; HI is the hazard index calculated by estimating the sum of the HQs; CR_i_ is the carcinogenic risk of the ith element attributed to all the pathways (19). The interpretation and values of the exposure parameters are shown in Supplementary Tables S1, S2. A value of HQ or HI of < 1 suggests that detrimental health effects are not possible in the exposed population; when HQ or HI is close to or over 1, the adverse health effects should receive more attention (22, 23). Cancer index values of heavy metals in the range of 10^−6^–10^−4^ are acceptable and do not pose a significant carcinogenic risk (3).

Probabilistic estimation was adopted to estimate the non-determinacy and variation in the risk assessment by the Monte Carlo simulation. The distribution of parameters (the concentrations of heavy metals, daily maize intake, exposure frequency, body weight, etc.) was determined according to the US EPA guidelines and the Chinese Exposure Parameters Guidebook. The process was performed with Oracle Crystal Ball software. The Monte Carlo simulation was run for 10,000 iterations with 95% confidence level by randomly sampling values from the distribution of the exposure parameter. The population distribution of health risks was derived. A sensitivity analysis was performed by Crystal Ball (Oracle, Redwood City, CA, USA) to verify the contribution of each variable to the health risk model.

### Data analysis

Descriptive statistical analysis was performed to calculate the characteristics of the heavy metal contamination in soil and maize in Microsoft Excel 2016 (Microsoft Corporation, Redmond, USA); data are presented as means ± standard deviations. To compare the heavy metal pollution of soil and maize in the control and polluted areas, comparisons of two means were analyzed by a *t*-test, and multiple comparisons were analyzed by one-way analysis of variance (ANOVA) followed by the Student–Newman–Keuls (SNK) test with SPSS 22.0 (IBM Corp, Armonk, USA). The level of significance was set at 5%. Additionally, correlation analysis and random forest analysis were performed to describe the relationship between two or more heavy metal variables using R Studio (Integrated Development for R. RStudio, MA, USA). The determination of the best-fitting distribution, Monte Carlo simulation, and sensitivity analysis were carried out in Crystal Ball software. *P*-values < 0.05 were regarded as significantly different, and all *P*-values and 95% confidence intervals were two-tailed. All figures were constructed with Origin 2018 (Origin Lab, MA, USA).

## Results

### Basic characteristics of heavy metals in soil and maize

**Figure 2:**
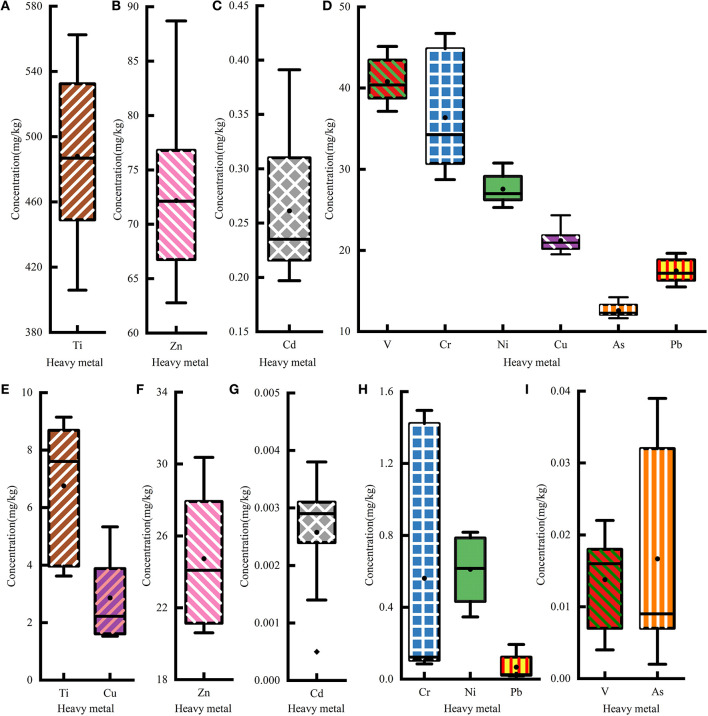
Statistics of the heavy metal concentration in agricultural soil and maize. The heavy metal concentrations of **(A–D)** soil and **(E–I)** maize.

Nine heavy metals (Cr, V, Ni, Pb, As, Ti, Cd, Cu, and Zn) were detected in all samples. The descriptive statistics of the studied heavy metals in soil and maize are summarized in Figure 2 and Table 1. In the soil samples, the mean concentrations of all heavy metals except V, Cr, and Ti were greater than their corresponding background levels (Table 1). According to the risk control standard of agricultural soil in China (GB15618-2018), none of the soil samples had heavy metals concentrations exceeding the screening value. The concentrations of soil heavy metal in the polluted areas were all greater than those in the control areas, except for the concentration of Hg.

A total of 77.78% of maize samples had Ni concentrations higher than the standard levels for corn in China (NY 861-2004). In addition, the mean concentrations of maize Ni were greater than the standard levels (NY 861-2004). The concentrations of all heavy metals in this study except V were greater than those in the CK area. This could indicate that most heavy metals (except V) accumulate in maize.

The analysis results indicate that heavy metals induced varying degrees of contamination in soil and maize in our study area; Cd was the major soil pollutant, and Ni was the main maize pollutant.

### Heavy metal pollution assessment of soil and maize

**Figure 3:**
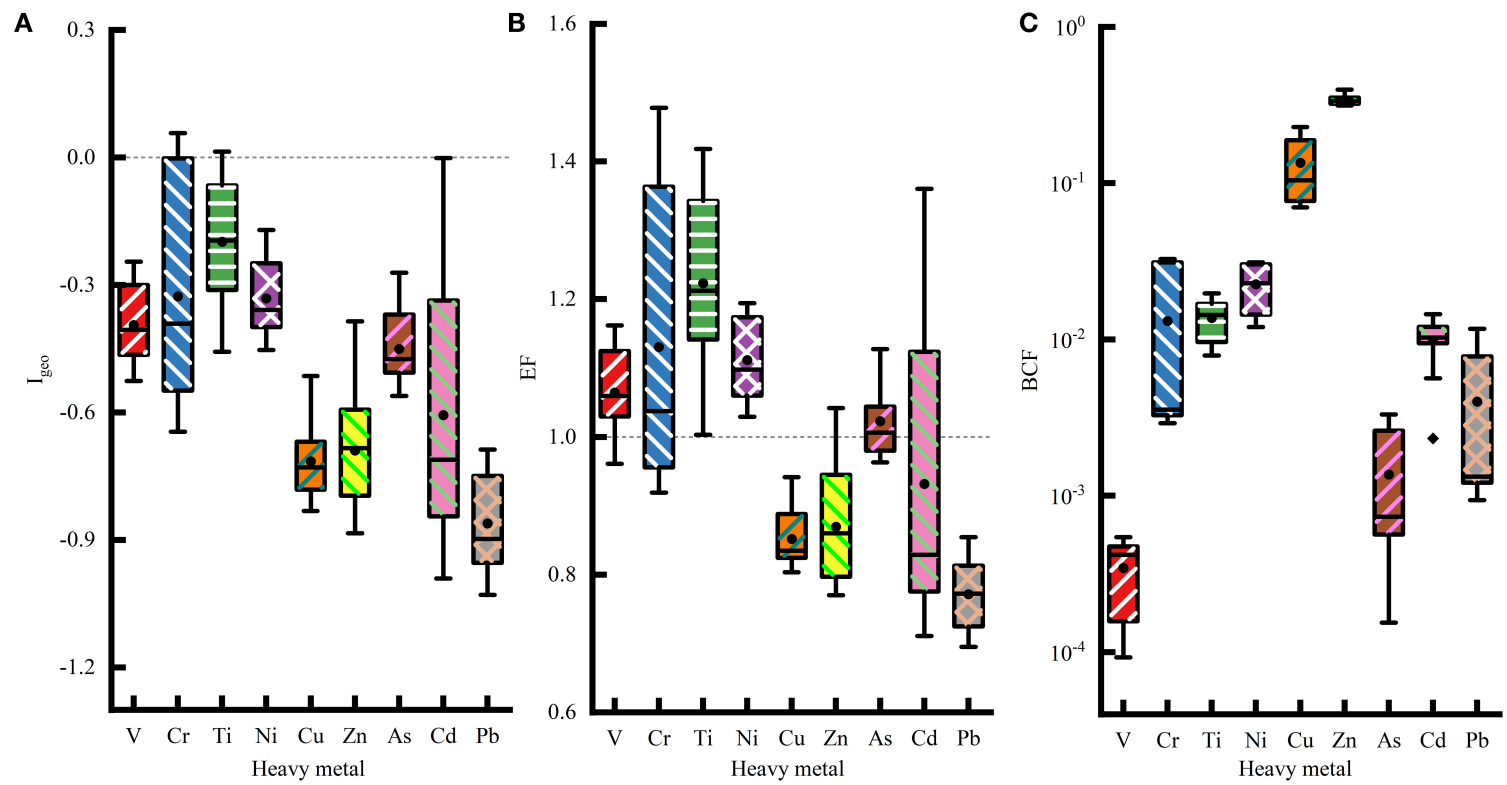
The pollution status assessment of soil and maize heavy metal. Geo-accumulation index (Igeo) **(A)** and enrichment factor (EF) **(B)** of soil, and bioaccumulation factor (BCF) **(C)** of maize for heavy metal pollution in the study area.

The I_geo_, EF, and BCF were used to assess the pollution level of the soil and maize heavy metal, and the results are shown in Figure 3. The I_geo_ and EF were evaluated to assess soil heavy metal contamination status. The mean I_geo_ values of all heavy metals in the sampling areas were smaller than 0, indicating unpolluted levels (Figure 3A). The mean I_geo_ of Cr in the TR area was higher than zero, indicating a slightly polluted level (Supplementary Figure S1A). The mean EF values of all heavy metals except for Zn, Cu, Cr, and Pb were higher than 1 (Figure 3B). Soil Pb had the lowest EF value (0.77) among the nine heavy metals. Soil V, Ti, and Ni were slightly enriched in all sampling areas. Soil Cr and Cd were slightly enriched in the TR area, and the soil Cr and As were slightly enriched in the CW area (Supplementary Figure S1B). Furthermore, soil V, Cr, Ti, and Ni had higher EF values than soil Cu, Zn, and Pb. Soil Ti was the most prevalent heavy metal in the study area and had the highest EF, followed by Cr. The EF values of Ti and Ni were higher than 1 in all samples.

The BCFs of all heavy metals in maize were < 1 (Figure 3C; Supplementary Figure S1C), which indicates that the physiological need for these elements was rather limited. In addition, the heavy metal pollution level was low. Zn had the highest BCF value, which indicates that it had the highest mobility in maize. Zn had the highest mean BCF values, followed by Ni, Cu, Ti, Cr, Mn, Cd, Pb, As, and V.

### Health risk assessment of soil heavy metal

The probabilistic health risk assessment indexes related with the three direct soil exposure pathways (dermal absorption, ingestion, and inhalation) were assessed by the hazard quotient (HQ), hazard index (HI), carcinogenic risk (CR), and total carcinogenic risk (TCR) with the Monte Carlo simulation (Table 2).

**Table 2:** Estimation of non-carcinogenic (Hazard quotient, HQ; Hazard index, HI) and carcinogenic risks (CR) posed by heavy metal in soil *via* Monte Carlo simulation.

**Population**	**V**	**Cr**	**Ni**	**Cu**	**Zn**	**As**	**Cd**	**Pb**	**Total**
**HQ-soil ingestion**									
Adults	2.09 × 10^−3^	5.72 × 10^−3^	6.36 × 10^−4^	2.45 × 10^−4^	1.11 × 10^−4^	1.94 × 10^−2^	1.22 × 10^−4^	2.32 × 10^−3^	3.06 × 10^−2^
Children	1.60 × 10^−2^	4.39 × 10^−2^	4.88 × 10^−3^	1.87 × 10^−3^	8.52 × 10^−4^	1.49 × 10^−1^	9.39 × 10^−4^	1.78 × 10^−2^	2.35 × 10^−1^
**HQ-dermal contact**									
Adults	4.73 × 10^−5^	1.29 × 10^−4^	1.44 × 10^−5^	5.54 × 10^−6^	2.52 × 10^−6^	1.32 × 10^−2^	2.77 × 10^−6^	5.25 × 10^−5^	1.34 × 10^−2^
Children	3.13 × 10^−4^	8.56 × 10^−4^	9.53 × 10^−5^	3.66 × 10^−5^	1.66 × 10^−5^	8.71 × 10^−2^	1.83 × 10^−5^	3.47 × 10^−4^	8.88 × 10^−2^
**HQ-soil inhalation**									
Adults	4.50 × 10^−7^	1.23 × 10^−6^	1.37 × 10^−7^	5.26 × 10^−8^	2.39 × 10^−8^	4.18 × 10^−6^	2.63 × 10^−8^	4.99 × 10^−7^	6.59 × 10^−6^
Children	8.98 × 10^−7^	2.46 × 10^−6^	2.73 × 10^−7^	1.05 × 10^−7^	4.78 × 10^−8^	8.33 × 10^−6^	5.26 × 10^−8^	9.96 × 10^−7^	1.32 × 10^−5^
**HI**									
Adults	2.14 × 10^−3^	5.85 × 10^−3^	6.51 × 10^−4^	2.50 × 10^−4^	1.14 × 10^−4^	3.26 × 10^−2^	1.25 × 10^−4^	2.37 × 10^−3^	4.41 × 10^−2^
Children	1.63 × 10^−2^	4.47 × 10^−2^	4.97 × 10^−3^	1.91 × 10^−3^	8.69 × 10^−4^	2.36 × 10^−1^	9.57 × 10^−4^	1.81 × 10^−2^	3.24 × 10^−1^
**CR-soil ingestion**									
Adults	–	2.41 × 10^−6^	6.05 × 10^−6^	–	–	2.45 × 10^−6^	2.14 × 10^−7^	1.93 × 10^−8^	1.11 × 10^−5^
Children	–	4.60 × 10^−6^	1.16 × 10^−5^	–	–	4.66 × 10^−6^	4.08 × 10^−7^	3.68 × 10^−8^	2.13 × 10^−5^
**CR-dermal contact**									
Adults	–	5.49 × 10^−8^	1.38 × 10^−7^	–	–	5.57 × 10^−8^	4.88 × 10^−9^	4.39 × 10^−10^	2.54 × 10^−7^
Children	–	9.09 × 10^−8^	2.29 × 10^−7^	–	–	9.23 × 10^−8^	8.08 × 10^−9^	7.28 × 10^−10^	4.21 × 10^−7^
**CR-soil inhalation**									
Adults	–	5.20 × 10^−10^	1.30 × 10^−9^	–	–	5.27 × 10^−10^	4.61 × 10^−11^	4.15 × 10^−12^	2.40 × 10^−9^
Children	–	5.49 × 10^−8^	1.38 × 10^−7^	–	–	5.57 × 10^−8^	4.88 × 10^−9^	4.39 × 10^−10^	2.54 × 10^−7^
**TCR**									
Adults	–	2.46 × 10^−6^	6.19 × 10^−6^	–	–	2.50 × 10^−6^	2.19 × 10^−7^	1.97 × 10^−8^	1.14 × 10^−5^
Children	–	4.74 × 10^−6^	1.19 × 10^−5^	–	–	4.81 × 10^−6^	4.21 × 10^−7^	3.80 × 10^−8^	2.19 × 10^−5^

**Figure 4:**
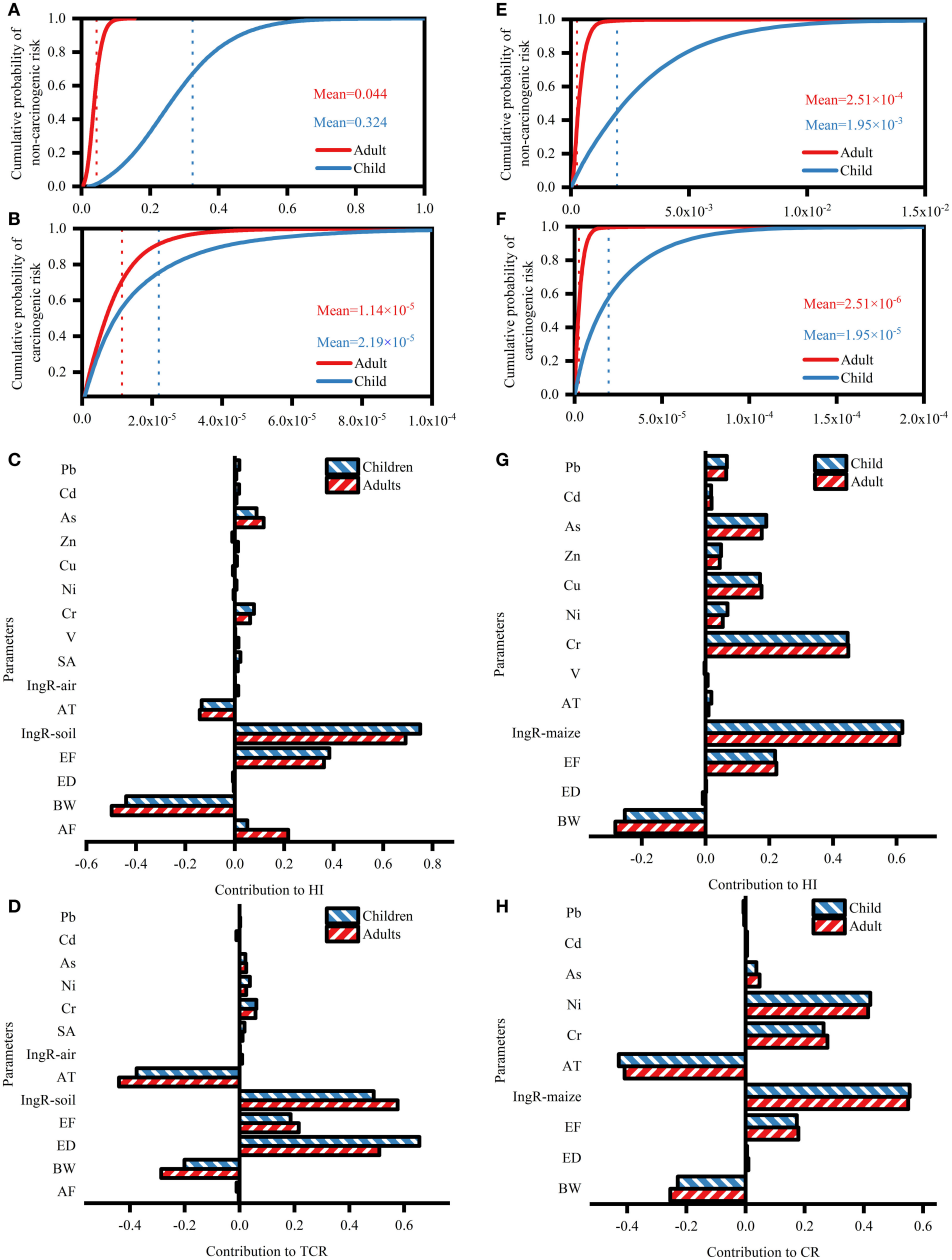
The health risk assessment of heavy metal. **(A)** Probability distribution of hazard index (HI) and **(B)** total cancer risk (CR) in soil. (the blue or red dashed vertical lines presented the mean values for adults and children); **(C)** Contribution of different exposure parameters to hazard index (HI) and **(D)** total cancer risk (TCR) in soil; **(E)** Probability distribution of hazard index (HI) and **(F)** total cancer risk (CR) in maize. (The blue or red dashed vertical lines presented the mean values for adults and children); **(G)** Contribution of different exposure parameters to hazard index (HI) and **(H)** total cancer risk (TCR) in maize.

The non-carcinogenic health risks were only estimated for Cr, V, Ni, Pb, As, Cd, Cu, and Zn because Ti lacked a reference exposure dose (Table 2). In children and adults, the mean HQ values of the eight heavy metals evaluated were lower than the risk threshold of 1. Particularly, the HQ value of As was at least an order of magnitude greater than values of the other heavy metals. The mean HI values were both lower than 1 (3.24 × 10^−1^ and 4.41 × 10^−2^ for children and adults, respectively; Figure 4A). These results indicate a low probability of the occurrence of adverse health effects caused by the soil. Furthermore, the non-carcinogenic health risk to children was 7.35 times more severe than the risk to adults, indicating that children had a far higher chance of non-carcinogenic health consequences due to heavy metal exposure. For children and adults, soil ingestion posed the greatest health risk, followed by dermal absorption and inhalation (Table 2). As posed the highest total non-carcinogenic health risk (THQ) for adults and children, followed by Cr, Pb, V, Ni, Cu, Cd, and Zn. Specifically, As and Cr accounted for 73.92 and 13.27% of the HI for adults and 72.84 and 13.80% of the HI for children, respectively. Approximately 0.02% of all children had HI values > 1, and none of the adults had an HI > 1, suggesting a low non-carcinogenic health risk.

The CR was only estimated for Cr, Ni, As, Cd, and Pb because Zn, Ti, Cu, and V lacked carcinogenic slope factors (Table 2). The mean CR values of the five heavy metals evaluated were below the acceptable level of 1 × 10^−4^. CR value of Ni contributed most to TCR, accounting for nearly 54.17 and 54.50% for children and adults, respectively. Ingestion was associated with the highest CR, followed by dermal contact and inhalation. The total CR of children was 7.77 times higher than that of adults. Notably, the CR value for heavy metal exposure *via* respiratory inhalation in children was approximately 106 times higher than that in adults. The mean TCR values were 1.14 × 10^−5^ and 2.19 × 10^−5^ in children and adults, respectively, within the acceptable range of 1 × 10^−6^ to 1 × 10^−4^ (Figure 4B). Approximately 0.02% of all adults and 1.14% of children had HI values > 10^−4^. Therefore, the CR differed between age groups. In general, the risks of soil heavy metals to human health were tolerable, and might be close to the acceptable limit.

The sensitivity analyses to TCR and HI showed of the exposure parameters, oral ingestion rate of soil dust contributed the most to the HI of adults and children with correlation coefficients of 0.75 and 0.69, respectively (Figure 4C). The content of soil As contributed the most to the HI among these heavy metals (correlation coefficients of 0.12 in adults and 0.09 in children). Of the exposure parameters, the oral ingestion rate of soil dust contributed the most to CR in adults, followed by exposure duration; the correlation coefficients for soil ingestion rate and exposure duration were 0.58 and 0.50, respectively (Figure 4D). For children, the order was the opposite: exposure duration had the greatest contribution, followed by the soil ingestion rate. The Cr content in the soil contributed the most to the TCR among all heavy metals. Body weight and average exposure time showed a negative impact on the HI and TCR of inhabitants.

### Health risk assessment of maize heavy metal

A comparison of non-carcinogenic human health risks between soil and maize exposure is provided in Table 3. In children and adults, the mean HQ values of the eight heavy metals evaluated for maize were lower than 1; Cr had the greatest HQ value, followed by Cu, Zn, As, Ni, Pb, Cd, and V. The mean HI values of children and adults *via* maize consumption were 2.51 × 10^−4^ and 1.95 × 10^−3^, respectively (Figure 4E). Adults and children exhibited a lower risk from maize consumption than from soil exposure. Children were 7.77 times more likely to face serious risk from maize heavy metal pollution than adults. None of the HI for all inhabitants were more than 1, suggesting no non-carcinogenic health risk.

**Table 3:** Estimation of non-carcinogenic (Hazard quotient, HQ; Hazard index, HI) and carcinogenic risks (CR) posed by heavy metal in maize *via* Monte Carlo simulation.

**Population**	**V**	**Cr**	**Ni**	**Cu**	**Zn**	**As**	**Cd**	**Pb**	**Total**
**HQ**									
Adults	8.96 × 10^−7^	1.20 × 10^−4^	1.33 × 10^−5^	3.98 × 10^−5^	3.65 × 10^−5^	3.02 × 10^−5^	1.12 × 10^−6^	1.01 × 10^−5^	2.51 × 10^−4^
Children	6.88 × 10^−6^	9.29 × 10^−4^	1.03 × 10^−4^	3.12 × 10^−4^	2.81 × 10^−4^	2.33 × 10^−4^	8.58 × 10^−6^	7.83 × 10^−5^	1.95 × 10^−3^
**CR**									
Adults	–	6.81 × 10^−7^	1.74 × 10^−6^	–	–	5.17 × 10^−8^	2.70 × 10^−8^	1.19 × 10^−9^	2.51 × 10^−6^
Children	–	5.59 × 10^−6^	1.33 × 10^−5^	–	–	3.96 × 10^−7^	2.08 × 10^−7^	8.95 × 10^−9^	1.95 × 10^−5^

The health risk results of maize consumption revealed no carcinogenic effects of Cr, Ni, As, Cd, or Pb. The cancer risk of Ni was the greatest among these heavy metals *via* maize ingestion within an acceptable range for all inhabitants. The CR of Ni contributed the most to the TCR, with a contribution of 69.32% for adults and 68.21% for children, respectively. The mean TCR values and *via* maize consumption were 2.51 × 10^−6^ for adults and 1.95 × 10^−5^ for children, respectively (Figure 4F). Approximately 0.02% of adults and 1.44% of children had HI values > 10^−4^. Thus, the CR was greater for children than for adults. The overall risk from soil exposure was higher than that from maize consumption (4.54 times for adults and 1.12 times for children).

The results of the sensitivity analysis revealed that the consumption of maize was the most sensitive parameter for human health risk, with a contribution of 60.85% for adults and 61.82% for children to non-carcinogenic health risk (Figure 4G) and a contribution of 54.95% for adults and 55.46% for children to CR (Figure 4H). The second most sensitive parameter in the non-carcinogenic health risk assessment was the maize Cr concentration (44.86% contribution for adults and 44.62% contribution for children). The second most sensitive parameter in the carcinogenic risk assessment was the concentration of Ni in maize (41.42% contribution for adults and 42.15% contribution for children). Body weight showed a negative impact on the HI and TCR in all inhabitants. Furthermore, the average exposure time negatively affected the TCR, with a higher contribution than body weight.

### Relationship between heavy metal concentrations in the soil and maize

**Figure 5:**
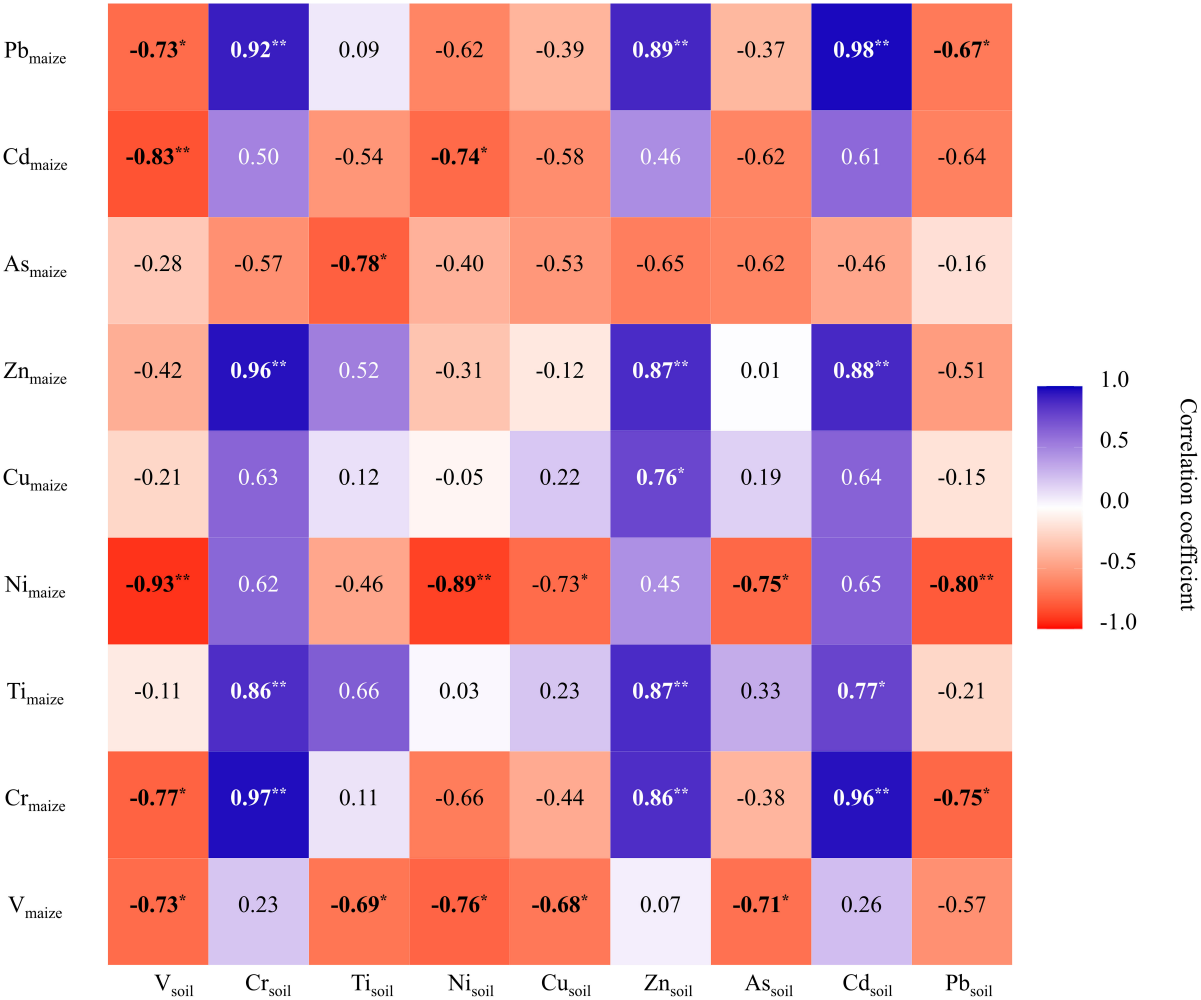
Correlations between heavy metal concentrations in maizes and soil.

Strong correlations were identified between the heavy metal concentrations of soil and maize. The Pearson correlation analysis results are shown in Figure 5. Overall, maize Ni, Pb, and Cr were affected the most by soil heavy metals, followed by V and Ti. Specifically, the maize Zn, Pb, and Cr concentrations showed a very significant positive correlation with Cr, Zn, and Cd concentrations in soil. Furthermore, maize Pb and Cr concentrations were significantly negatively correlated with soil V and Pb concentrations, and the maize Ni concentration showed a very significant negative correlation with soil Pb, Ni, and V. Moreover, the concentration of maize Ni showed a very significant negative correlation with soil Ni. By contrast, maize Cd showed a very significant negative correlation with soil V. The concentration of maize Ti showed a very significant positive correlation with soil Zn and Cr.

**Figure 6:**
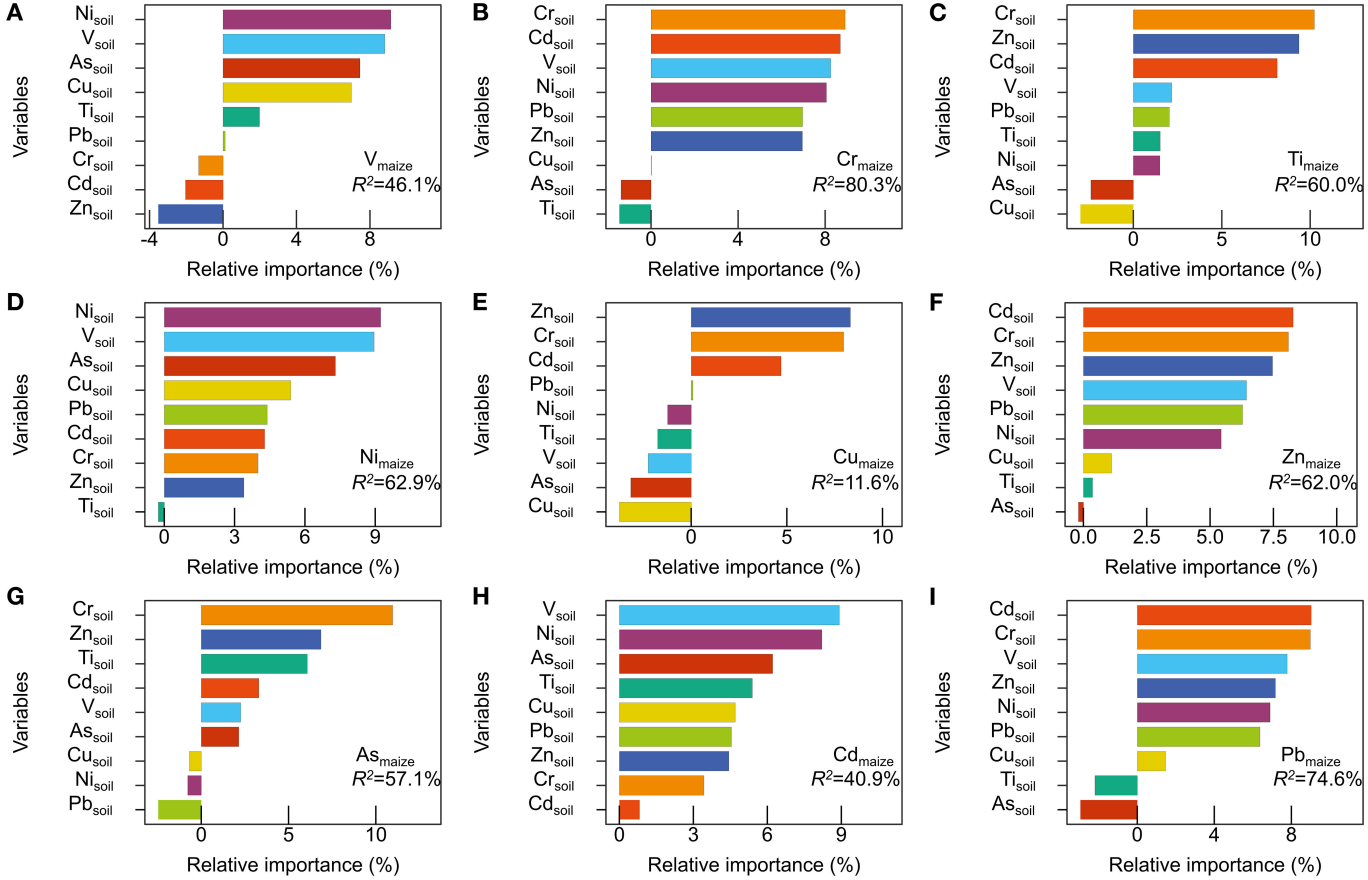
Plots of the variable importance measures of maize heavy metal. Maize heavy metal **(A)** V; **(B)** Cr; **(C)** Ti; **(D)** Ni; **(E)** Cu; **(F)** Zn; **(G)** As; **(H)** Cd; and **(I)** Pb was predicted by nine soil heavy metals.

The relative importance of the nine soil heavy metals to the nine maize heavy metals was determined by random forest models (Figure 6). The concentrations of soil Cr, Cd, and V were the factors with the highest influence on the concentration of the nine maize heavy metals. Compared with other heavy metals in maize, soil Cr contributed more to maize Cr, Cu, Ti, As, Pb and Zn; soil Cd contributed more to Cu, Cr, Ti, Pb, and Zn in maize; soil V contributed more to Cr, V, Ni, Cd, and Pb in maize. The three most crucial factors explaining the maize V, Ni, and Cd contents were soil Ni, As, and V; the three most crucial factors explaining the maize Cr and Pb contents were soil Cr, Cd, and V; and the three most crucial factors explaining the maize Ti, Zn, and Cu contents were soil Cr, Zn, and Cd. Finally, the three most crucial factors explaining the maize As content were soil Ti, Zn, and Cr.

## Discussion

### Assessment of heavy metal pollution in soil and maize

This study demonstrated that the pollution of soil and maize heavy metals, especially Ni, differed in the coal mining area. The mean concentrations of soil Ni, Ti, Cd, Cu, Pb, As, Sn, Hg, and Zn were all greater than the local background levels in Shanxi Province, but the values did not exceed the screening values (GB15618-2018). Notably, only the Ni concentration of maize was higher than the screening value (NY 861-2004), which might be due to the higher capacity of maize to attract soil Ni (17). Furthermore, some researchers have reported that atmospheric deposition, such as coal dust, and polluted irrigation water, such as wastewater from coal washing plants, may also increase the maize Ni (24, 25). Coal dust is generated in the process of coal mining and transportation and can readily diffuse into the atmosphere and be deposited onto the surface of corn (26). Wastewater from coal mine processing around the investigated areas was positively correlated with the accumulation of maize Ni (27). Additionally, because Ni is a key soil heavy metal contaminant in the agricultural soil, higher Ni mobility might cause abnormal growth and development in the maize system (28). It is necessary to reverse Ni pollution in the soil to eliminate its risks to the ecosystem and human health.

The I_geo_, EF, and BCF indices were evaluated to the contamination of the heavy metals in soil and maize. The mean I_geo_ of all heavy metals in the sampling areas was < 0, which indicated low geochemical contribution to these heavy metals in the cultivated soil (20). The mean EF values of V, Cr, Ti, Ni, and As were higher than 1, suggesting the enrichment of these heavy metals. Specifically, the EFs of Cr and Cd in the TR area fell into the significantly enriched range, which suggests that this region had substantial Cr and Cd inputs (1). On a large farm in Ghana, the enrichment of Cr and Ni was greater than that of Zn, Hg, Cd, and Fe (20). In our study, V, Cr, Ti, and Ni had high enrichment (EFs > 1), consistent with the results of Affum et al. (20). The BCFs of all heavy metals in maize were < 1, demonstrating that maize's physiological need for these heavy metals was rather limited (29). A higher BCF indicates a stronger ability of maize to attract heavy metals from the soil and an inferior capability to retain heavy metals (15). In our study, Zn and Cu had the highest BCFs, which implies that maize can store Zn and Cu more easily than other heavy metals (30).

### Probabilistic health risk assessment of soil heavy metal

The hazard index values of all heavy metals in the soil of the study area were lower than 1, indicating no significant non-carcinogenic health risk (31). The hazard quotient of As was more than an order of magnitude greater than those of other heavy metals. In Panzhihua City, the non-carcinogenic health risk of soil As was the greatest among all heavy metals, with an HQ > 1 (32). Although As had a lower concentration in crops than other heavy metals in Hamadan and a lower reference dose, the As concentration was far above the tolerable limit (8). The total non-carcinogenic health risk of children was 7.35 times more severe than that of adults, which indicates that children were more impressionable to soil heavy metal contamination, possibly because of their frequent hand-to-mouth behavior and higher respiration rate per unit of body weight (19).

The total carcinogenic risk values for all inhabitants fell within the acceptable range of carcinogenic risk; similar results were found in a study by Chen et al. (19). The concentration of soil Ni had the greatest average CR value. Along the South China coast, soil Ni also had the highest CR value compared with As, Cd, and Cr (33). Cancer risk was largely attributed to Ni, which accounted for 54.17 and 54.50% of TCR (33). In addition, long-term environmental exposure to Ni was reported to be correlated with an growing risk of gastrointestinal cancer (34). The high CR in children might be related to frequent hand-to-mouth behavior; the relationship between their exposure and body size; their developing body; or their poor ability to metabolize and excrete toxins (8). In our study, the pathway of oral ingestion contributed most to human health risks. The results of research from Sialkot in Punjab, Pakistan, showed that the ingestion was a major contributor to TCR, followed by dermal and inhalation pathways (31).

The sensitivity analysis of health risk assessment showed that the contributions of soil As and Cr concentrations were significantly greater than those of other heavy metals; As and Cr had a strong impact on the potential non-carcinogenic health risk and carcinogenic health risk in all populations, respectively. However, the most influential factors of soil health risk were soil ingestion rate and exposure duration, whereas body weight and average exposure time had a negative influence. Consistent with our results, Kharazi et al. (8) supported that the soil ingestion rate and exposure duration most easily affected the risk assessment of different populations, and body weight was a sensitive parameter for CR with a negative correlation. In probabilistic health risk research conducted by Wen'ling, body weight showed a negative impact on the HI and TCR of heavy metal exposure (19).

In conclusion, the non-carcinogenic and carcinogenic risks were all within the acceptable range, but the exposure risk of soil As and Ni among all heavy metals contributed most to the non-carcinogenic and carcinogenic risks, respectively. Therefore, the monitoring of As and Ni in soils needs to be strengthen to prevent health risks. Soil ingestion was identified as the most crucial exposure pathway for soil heavy metals (31). The reduction of health risk by soil ingestion could be bought about by reducing human interaction with the soil and farming time; this could be accomplished with the automation of farming practices, such as wireless communications, machine learning, artificial intelligence, and deep learning (35).

### Probabilistic health risk assessment of maize heavy metal

The potential non-carcinogenic health risk and carcinogenic risk of maize heavy metals fell within the acceptable range; these results were consistent with those of Liu et al. (11). The concentrations of maize Cr and Ni were the major contributors to non-carcinogenic health risk and carcinogenic risk, respectively. Taiwo et al. (14) reported that Cr had the largest HQ of all metals, with an HQ > 1 in the maize samples, and the carcinogenic evaluation of Ni and Cr in crops showed CR values above the acceptable threshold of 1.0 × 10^−4^. Children were the most sensitive population in terms of the non-carcinogenic health risk and carcinogenic risk of consuming maize in the contaminated area. Because maize is the residents' major food, it might contribute to the accumulation of heavy metals and heavy metal poisoning, especially in pregnant people and children (13).

Regarding the sensitivity analysis of health risk assessment, the consumption of maize was the most sensitive parameter for human health risk. These results are similar to those from research on heavy metals in agricultural soil and food crops in Hamadan, Iran (8). The contribution of maize Cr and Ni concentrations was significantly higher than that of other heavy metals in the assessment of non-carcinogenic health risk and carcinogenic risk, respectively. These results imply that controlling the consumption of maize and monitoring maize heavy metals, especially Cr, As, and Ni, could effectively reduce the health risk for residents.

### Relationship between heavy metal concentrations in the soil and maize

In this study, correlation analysis and random forest analysis demonstrated that the concentrations of soil Cr, V, and Cd could contribute more to the absorption of heavy metals by maize. Soil Cr increased the absorption of Cr, Ti, Cu, Zn, and Pb by maize and decreased the absorption of maize As, suggesting that soil Cr could accelerate the crop assimilation of Ti, Cr, Cu, Zn, and Pb. Xiang et al. found that the assimilation of Zn by crops was affected by the synergistic effect of Cr in soil (17), which supports the findings of the present study. Huang et al. (30) reported that the concentrations of Cr and Ni in dryland soil were positively correlated with the concentrations of heavy metals in corresponding crops in Hunan Province, China. Soil Cd was positively correlated with maize Cd, but the correlation was not significant. A study by Wang et al. (36) found that different Cd sources, such as irrigation, fertilization, manure fertilizer, and atmospheric deposition, had a strong influence on the uptake of Cd by rice. Additionally, the assessment of heavy metal contamination of maize should consider the heavy metal availability instead of only relying on the total soil heavy metal concentrations (7). A change in the soil's physical and chemical properties could be one of the reasons for the lower mobility and bioavailability of heavy metals, which could lead to higher heavy metal concentrations (37). Soil Ni contributed negatively to the absorption of V, Ni, and Cd by maize. Using Pearson correlation analysis, Huang and Gui observed a negative correlation between Ni in soil and Cd in maize grain parts (38). The concentrations of soil V and Ni had a very significant negative contribution to the absorption of corresponding heavy metals by maize, indicating that the pollution of soil V and Ni might not be the main source in maize and there might be other pollution sources (38). The establishment of random forest models would be helpful to preliminarily predict the concentrations of these heavy metals and contribute to quantitatively and comprehensively assessing the ecological and health risks of heavy metals in maize.

## Conclusion

Soil heavy metals posed severe risks to human health through the food chain. This study intended to investigate the heavy metal content of soil and maize, assess health risks and explore the relationships between heavy metals in the soil and maize, to provide support for early warning of human health risks. Although the average soil heavy metal concentrations did not exceed the national standards, the average concentrations of maize Ni exceeded the standards for food. The health risks for nearly all maize and soil heavy metals were low. The soil As and Ni concentrations contributed the most to non-carcinogenic and carcinogenic risks, respectively, and maize Cr and Ni contributed the most to non-carcinogenic and carcinogenic risks, respectively. Furthermore, the results showed that maize heavy metals were influenced the most by Cr, Cd, and V in the soil. Further studies are recommended to explore the transfer mechanism of Ni between soil and maize and regulate the Ni content in maize within an appropriate range. The significance of limiting Ni concentrations in the maize to 0.40 mg/kg is highlighted. This study shaded light on heavy metal pollution and build on previous by providing detailed information on maize.

The Monte Carlo method was employed for probabilistic estimation of health risks, taking into account the variability of exposure parameters and the uncertainty of heavy metal concentrations, which made the results more reliable. Whereas, the health risk of crop heavy metal in this study was assessed only for maize as the representative crop in the investigated areas, but did not consider any other crops, such as rice, wheat and vegetables. To comprehensively estimating human health risks in the future research, detailed consumption lists should be developed to quantify sources of heavy metals. The random forest model was used for assessing the inner relationship of pollution risk in soil-crop system, revealing the complex relationships between soil and maize heavy metals. However, the random forest model could not reveal the dynamic changes in heavy metal concentrations between soil and maize. Therefore, it is necessary to explore the dynamic process of heavy metal transfer from soil to crops.

## Data availability statement

The original contributions presented in the study are included in the article/Supplementary material, further inquiries can be directed to the corresponding authors.

## Author contributions

XY and BC: conceptualization, methodology, software, validation, formal analysis, data curation, and visualization. XY, YG, and HZ: investigation. HZ and LL: resources and supervision. BC: writing—original draft preparation. XY and LL: writing—review and editing. LL: project administration. XY: funding acquisition. All authors have read and agreed to the published version of the manuscript.

## Funding

This work was jointly supported by Scientific and Technological Innovation Programs of Higher Education Institutions in Shanxi (Grant No. 2021L222), Teaching Reform Innovation Programs of Higher Education Institutions in Shanxi (Grant No. J20220435), the Applied Basic Research Foundation of Shanxi Province (Grant No. 201801D221404), and the Startup Foundation for Doctors of Shanxi Medical University (Grant No. 03201522).

## Conflict of interest

The authors declare that the research was conducted in the absence of any commercial or financial relationships that could be construed as a potential conflict of interest.

## Publisher's note

All claims expressed in this article are solely those of the authors and do not necessarily represent those of their affiliated organizations, or those of the publisher, the editors and the reviewers. Any product that may be evaluated in this article, or claim that may be made by its manufacturer, is not guaranteed or endorsed by the publisher.
