# Supplementary material for: Heavy metal contamination assessment and probabilistic health risks in soil and maize near coal mines
Source: Front Public Health. 2022 Oct 13;10:1004579. doi: 10.3389/fpubh.2022.1004579 (PMC9606824; doi:10.3389/fpubh.2022.1004579)
Supplement: Supplementary file 1 [file Data_Sheet_1.docx]

Supplementary Material

# Supplementary Figures and Tables

## Supplementary Figures





**Supplementary Figure 1.** Geo-accumulation index (Igeo) and enrichment factor (EF) of soil, and bioaccumulation factor (BCF) of maize for the heavy metals in the three sampling areas.

## Supplementary Table

**TABLE S1 | The definitions and values of the intake exposure parameters**

| Parameter | Definition | Adult | Child | Unit |
| --- | --- | --- | --- | --- |
| C_i_ | Concentrations of heavy metals in soil | Our work | | mg/kg |
| IngR | Ingestion rate of soil | 100 | 200 | mg/day |
|  | Inhalation rate of soil | 14.7 | 7.63 | m^3^/day |
| CF | Conversion factor | 10^-6^ | | kg/mg |
| EF | Exposure frequency | 180 | | day/year |
| ED | Exposure duration | 24 | 6 | year |
| SA | Surface area of exposed skin | 5700 | 2800 | cm^2^ |
| AF | Skin adherence factor of soil | 0.2 | 0.7 | mg/(cm^2^•day) |
| ABS | Dermal absorption factor | 0.03 for As and 0.001 for all other | | unitless |
| BW | Body weight of exposed individual | 62 | 15.9 | kg |
| AT | Average exposure time | Non-carcinogenic effect:：AT = ED×365 Carcinogenic effect: AT = 70×365 | | day |
| PEF | Particle emission factor | 1.36×10^9^ | | m^3^/kg |

**TABLE S2 | The definitions and values of the health risk exposure parameters**

| Parameter | Definition | Element | Value | Unit |
| --- | --- | --- | --- | --- |
| RfD | Reference dose | V | 9×10^-3^ | mg/(kg·d) |
|  |  | Cr | 3×10^-3^ |  |
|  |  | Mn | 0.1 |  |
|  |  | Ni | 0.2 |  |
|  |  | Cu | 4×10^-2^ |  |
|  |  | Zn | 0.3 |  |
|  |  | As | 3×10^-4^ |  |
|  |  | Cd | 1×10^-3^ |  |
|  |  | Hg | 3×10^-4^ |  |
|  |  | Tl | 3×10^-6^ |  |
|  |  | Pb | 3×10^-3^ |  |
| SF | Cancer slope factor | Cr | 0.5 | kg/(d·mg) |
|  |  | Ni | 1.7 |  |
|  |  | As | 1.5 |  |
|  |  | Cd | 6.3 |  |
|  |  | Pb | 8.5×10^-3^ |  |
